# Exploring physician engagement in health care organizations: a scoping review

**DOI:** 10.1186/s12913-023-09935-1

**Published:** 2023-09-26

**Authors:** Anna Prenestini, Rocco Palumbo, Roberto Grilli, Federico Lega

**Affiliations:** 1 Department of Economics, Management and Quantitative Methods (DEMM) and Center of Research and Advanced Education in Health Administration (CRC HEAD), Università Degli Studi Di Milano, Milan, Italy; 2 Department of Management & Law, Università Degli Studi Di Roma Tor Vergata, Rome, Italy; 3 Health Services Research, Evaluation and Policy Unit, Local Health Authority of Romagna, Ravenna, Italy; 4 Department of Biomedical Sciences for Health (SCIBIS) and Center of Research and Advanced Education in Health Administration (CRC HEAD), Università Degli Studi Di Milano, Milan, Italy

**Keywords:** Physician, Clinician, Engagement, Health care organization, Scoping review, Performance, Quality

## Abstract

**Rationale:**

Enhancing health system effectiveness, efficiency, and appropriateness is a management priority in most world countries. Scholars and practitioners have focused on physician engagement to facilitate such outcomes.

**Objectives:**

Our research was intended to: 1) unravel the definition of physician engagement; 2) understand the factors that promote or impede it; 3) shed light on the implications of physician engagement on organizational performance, quality, and safety; and 4) discuss the tools to measure physician engagement.

**Method:**

A scoping review was undertaken. Items were collected through electronic databases search and snowball technique. The PRISMA extension for Scoping Reviews (PRISMA-ScR) statement and checklist was followed to enhance the study replicability.

**Results:**

The search yielded 16,062 records. After an initial screening, 300 were selected for potential inclusion in this literature review. After removing duplicates and records not meeting the inclusion criteria, full-text analysis of 261 records was performed, yielding a total of 174 records.

**Discussion:**

Agreement on the conceptualization of physician engagement is thin; furthermore, scholars disagree on the techniques and approaches used to assess its implementation and implications. Proposals have been made to overcome the barriers to its adoption, but empirical evidence about implementing physician engagement is still scarce.

**Conclusions:**

Our scoping review highlights the limitations of the extant literature about physician engagement. Physician engagement is a relatively ill-defined concept: developing an evidence base for its actual implementation is necessitated to provide reliable guidance on how the governance of health care organizations could be improved. Although we did not assess the quality or the robustness of current empirical research, our findings call for further research to: 1) identify potential drivers of physician engagement, 2) develop dependable assessment tools providing health care organizations with guidance on how to foster physician engagement, and 3) evaluate engagement’s actual impact on health care organizations’ performance.

**Supplementary Information:**

The online version contains supplementary material available at 10.1186/s12913-023-09935-1.

## Introduction

Health care institutions face significant challenges in enhancing their efficiency and effectiveness in delivering high-quality services [1]. One of the most pressing management issues faced by health care institutions is how to steer professional behavior toward organizational objectives [2, 3]. This issue is especially challenging for physicians, who are exposed to role hybridity and perceive the need to align their professional identity with contributing to the organization’s financial and economic viability. Davies et al. [4] argued that physicians are primarily loyal to patients and specialization and commit to the organization only secondarily. They are predominantly focused on patient health, treatment effectiveness, and evidence-based practice, and are less predisposed to embracing their organization’s strategic and operative goals [5]. Hence, physicians expect discretion when performing their work, with autonomy being a core principle reflected in the predominance of individual/group interests over organizational concerns [6].

Various initiatives can be taken to maintain hierarchical control over physicians’ behaviors and restrain the divergence of professional and organizational aims. Among others, economic incentives promoting organizational goals, information disclosure about service quality [7], and health technology assessment recommendations guiding the allocation of resources [8] entail attempts to narrow down physicians’ self-determination. These initiatives produce adverse effects due to behavioral distortion and performance management risks, such as gaming the system, ossifying and converting slacks into targets, biasing information [9], and fluctuating outcomes [10, 11]. However, they have proven helpful in directing focus on performance management and aligning professional behaviors with adherence to health care quality, efficiency, and effectiveness. They can be measured with specific indicators, whereas other relevant performance aspects are too complex to describe with indicators [12, 13].

Nevertheless, these initiatives are inevitably not decisive since most professionals’ decisions cannot be monitored, controlled, and described using precise process or outcome metrics. At the same time, there are promising studies on the influence of task uncertainty (with indicator controllability as a prerequisite) on the choice between process and outcome indicators [14]. Such limitation disrupts the link between methods to control professionals’ actions and organizational behaviors. The underlying problem remains how to steer professionals toward achieving organizational goals, given that clinical behaviors determine health service quality and effectiveness. One way would be to focus more on the nature of the relationship between health care organizations and physicians, shedding light on the latter engagement in organizational dynamics.

Engagement implies involving physicians in their organizations’ decision-making processes [15, 16]. It is conducive to aligning the interests of physicians with organizational concerns, prompting them to take direct responsibility in the formulation and pursuit of organizational goals [17]. This consists with the dimensions of health care quality, safety, and appropriateness, through which physicians play a decisive role in building organizational excellence [18–20]. Like other management philosophies, engagement practices come and go, sometimes presented as a radical innovation and in other cases framing an incremental improvement of existing approaches. In many circumstances, the transition towards engagement is not supported by empirical evidence witnessing the improvement of health care organizations’ performance [21].

Despite these considerations, engagement represents a common approach in health care management. Over the last 30 years, physicians have played a central role in designing and implementing management practices and improving organizational performance [22–24]. Engaging physicians in managerial decision-making processes involves a variety of functions, such as:

- Fulfilling top management positions, *e.g.*, general manager and chief executive [22, 25, 26];
- Assuming executive responsibility in the middle line, *e.g.*, department manager [27, 28];
- Participating in executive boards and monitoring health care services quality and effectiveness to improve organizational processes [29];
- Developing procedures to incentivize health care professionals to implement new projects and foster organizational change and innovativeness [30, 31].

Engagement relies on physicians’ skills, knowledge, and experience, enabling them to contribute to planning and managing services and boosting organizational outcomes [18–20, 32] consistently with a clinical governance perspective [29, 33]. Engaging physicians implies a corporate culture that stimulates employees’ participation in taking responsibility for internal management issues and accountability toward external stakeholders.

## Study aims

The definition of physician engagement is elusive, preventing us from better understanding its contents and implications for theory and practice. This study attempts to fill this knowledge gap, extrapolating from the extant scientific debate guidance to conceptualize physician engagement and promote it in contemporary health care institutions. More specifically, our research questions were:

- How is the concept of physician engagement defined?
- What do we know about the factors that can promote or impede it?
- How does physician engagement relate to organizational performance, quality, and safety?
- Which tools can be used to measure physician engagement?

In answering these questions, we emphasized the distinguishing nature of engagement as compared with germane concepts, such as physician leadership and job engagement, shedding light on the steps which should be taken to engage physicians. Moreover, we carefully took into account the relevant overlap with the concept of clinician engagement, which refers to many different categories of health professionals (e.g., doctors, nurses, and other nonphysician clinicians). Addressing these questions helps us better understand whether physician engagement is a genuine innovation or old wine in new bottles. For this purpose, this study presents what is currently known about physician engagement, its constitutive elements, and how it can be applied to enhance organizational performance effectively.

## Materials and methods

We undertook a scoping review of the extant scientific debate to answer the research questions [34]. Scoping reviews have been widely used to profile scholarly knowledge about a substantive study domain and on broad issues rather than on a narrowly defined research question, which is typical of systematic reviews. This article specifically focused on physician engagement, which has been identified as a timely and relevant study domain [35]. Mapping the extant scientific landscape, this review enabled us to spot areas of agreement and disagreement, delivering a preliminary systematization of the scholarly debate and paving the way for an agenda for further developments [36, 37].

We identified several factors prompting physicians to address management issues, such as the focus on quality, the quest for efficiency, and the research of health services’ appropriateness. We assumed that a more precise definition of physician engagement could be obtained by identifying its dimensions and enabling and impeding factors. This could inform the arrangement of policies and practices to promote physicians’ involvement in management decisions and organizational processes [38].

**Fig. 1:**
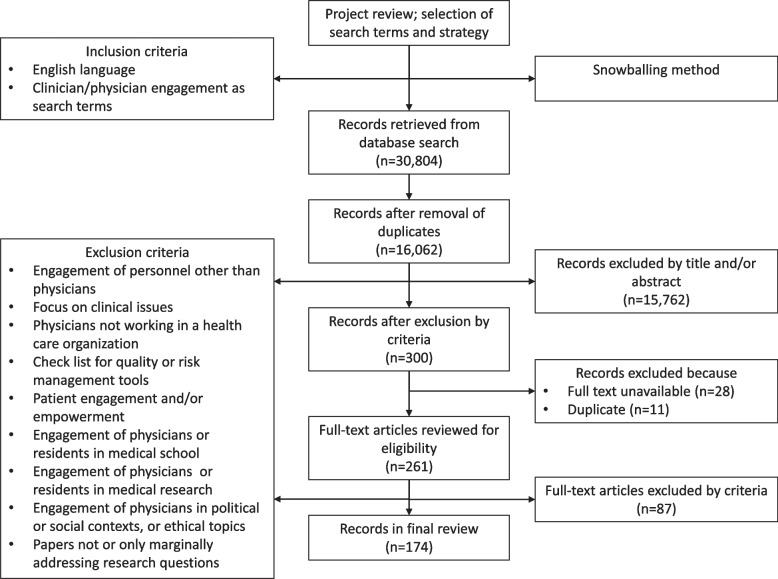
Study flow diagram

Arksey and O’Malley [36] and Levac et al. [37] describe the methodology framework used in this study. We followed the Preferred Reporting Items for Systematic Reviews and Meta-Analyses Extension for Scoping Reviews: PRISMA-ScR [39]. The checklist used to conduct this research is available in Supplementary Materials. The flow diagram illustrating this study is presented in Fig. 1.

### Step 1. Literature search

Multiple citation databases – Scopus, Embase, Web of Science, PubMed, and EBSCO Health Business Elite – were queried to collect relevant items for this literature review. Database search was subsidized with a snowball technique. This mixed approach enabled us to obtain a comprehensive overview of the current scholarly debate about physician engagement, contemplating both conventional academic publishing and grey literature. The following search terms were used to query citation databases:

“physician* engagement” OR “doctor* engagement” OR “medical engagement” OR “clinic* engagement” OR “engaging physician*” OR “engaging doctor*” OR “engaging medical” OR “engaging clinic*”

Studies published as of December 2020 were included in the analysis. This cut-off date was coherent with our aim to present a comprehensive and timely overview of studies on physician engagement, averting potential biases due to the COVID-19 pandemic. No other exclusion criteria were set regarding publication date, study design, and geographical origin, except for language. In fact, only items written in English were included in the review to permit the full replicability of our study protocol. Based on this search strategy, we retrieved 30,804 records. After the removal of duplicates, 16,062 items were processed in Step 2.

### Step 2. Exclusion criteria

A hybrid approach was taken to define inclusion criteria. *Ex-ante*, we embraced a deductive frame, drawing on the most impactful studies on physician engagement to determine the scope of our review. *In itinere*, we used an abductive approach based on the evidence collected from screened records to refine our review’s focus. Based on this routine, exclusion criteria were set using an iterative process involving two authors (AP and RG). Two other authors (FL and RP) confirmed the integration of these criteria with the initial exclusion criteria. More specifically, consistent with our focus on physician engagement, we decided to exclude the following:

- Studies on the engagement of nurses, other health care professionals, and senior or administrative staff;
- Studies on specific conditions and pathologies, therapies, and clinical trials;
- Studies on the engagement of physicians who were not involved in health care institutions (*e.g.*, general practitioners), for whom issues related to engagement are less relevant;
- Studies reporting checklists for measuring quality or patient safety;
- Studies dealing with the physicians’ participation in research, political activities, social initiatives, and ethical challenges in care delivery;
- Studies addressing patient engagement and investigating the professionals’ role in supporting patient empowerment;
- Studies focusing on physician engagement in medical schools and teaching institutions;
- Studies that did not focus on engagement as their primary concern.

Three authors (AP, RG, and RP) screened the records independently. The majority rule was adopted, *i.e.*, records were excluded by the agreement of two in three authors. A round of discussion was launched when needed. A fourth author (FL) was involved when no consensus could be reached. Starting from an initial database of 16,062 records, 15,762 were removed due to the application of the exclusion criteria reported above.

### Step 3. Record analysis

Of the 300 remaining records, 11 duplicate items were removed. Besides, 28 records were unavailable and, therefore, were retracted from the dataset. In sum, 261 records underwent full-text analysis; 87 were excluded because they were irrelevant to the study aims. Almost half of them did not comprehensively address physician engagement or marginally addressed our research questions (*n* = 46, 46.9%). Other records were discarded because they focused on technical tools for health care quality and safety (*n* = 9, 9.2%), on nonphysician roles (*n* = 8, 8.2%), on specific health conditions (*n* = 7, 7.1%), on patient empowerment- or engagement-related issues (*n* = 4, 4.1%), on medical research (*n* = 3, 3.1%), on political activities (*n* = 2, 2%), on contexts other than health care organizations (*n* = 5, 5.1%), or focused on medical school (*n* = 3, 3.1%).

Hence, 174 records were included in this scoping review (Appendix 1). The records were screened using an ad hoc classification technique. The items were examined for common elements, focusing both on the features of engagement (*e.g.*, characteristics of professionals and institutional attributes of health care organizations) and the rationale for engagement. Also, a description of the shades of engagement was rendered, looking at:

- 1. How the concept was operationalized in practice (*e.g.*, different types of clinical engagement);
- 2. How it was measured to assess the degree of engagement;
- 3. How it was related to organizational performance.

Lastly, we analyzed how physician engagement can be enhanced at the individual and the collective levels and which factors may promote or impede its implementation (*e.g.*, skills, competencies, and policies fostering employees’ participation). Studies exploring why engagement was successful and contributions (surveys, qualitative studies, *et similia*) reporting health professionals’ opinions and their attitudes toward physician engagement were precious for this purpose.

An electronic worksheet was created to standardize analysis and data collection. A training exercise on a random sample of 20 records was conducted by two authors (AP and RP) to define a coding strategy. Disagreements and improvements were discussed until consensus was achieved on the coding approach to classify selected items. One of the authors (AP) abstracted the data independently. Two authors (AP and RP) revised the results of the analysis. Disagreements were resolved through discussion with the other authors (FL and RG).

## Findings

### Overview

**Fig. 2:**
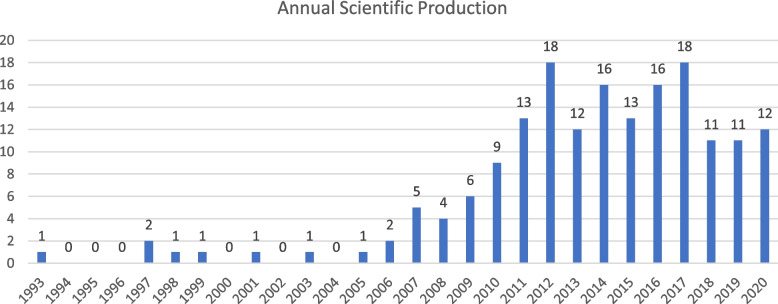
Number of papers by year of publication

Figure 2 displays the number of papers by year of publication. Ten studies about physician engagement were published between 1993 and 2006. Growth in the number of contributions started in 2007, with peaks in 2012 (publication of a series of reports and policy documents) and in 2017. A slight decrease was noticed between 2018 and 2020. Most studies reported cases in the UK, Canada, Australia, and northern Europe.

Table 1 presents the different types of publications: scholarly articles (*n* = 126; 72.4%), commentaries, perspectives, and editorials (*n* = 28; 16.1%) largely prevailed. Other publications included grey literature (*n* = 15; 8.6%), books (*n* = 4; 2.3%; one of which was a book chapter [40]), and a conference proceeding (*n* = 1; 0.6%). Most studies reported empirical research (*n* = 75; 43.1%) and conceptual advancements (*n* = 38; 21.8%). There were 26 (14.9%) position documents in the form of essays by influential scholars, opinion leaders, or organizations making recommendations to advance a topic. Alongside publications in academic journals, we included reports by The King’s Fund and other health care institutions, plus opinions/recommendations published in non-academic journals. There were 17 (9.8%) publications in non-academic journals, report studies, empirical research, or theoretical frameworks developed by scholars or scientific institutions. The search retrieved policy documents (*n* = 5; 2.9%), protocols (*n* = 2; 1.1%, one of which was a protocol for a scoping review [41]) and one a scoping analysis developed using mixed methods [42]. Nine (*n* = 9; 5.2%) studies did not fit any category.

**Table 1:** Study categories and types

**Document Category**	**n**	**%**
** Article**	126	72.4
** Perspective, editorial, letter, commentary**	28	16.1
** Reports/Gray literature**	15	8.6
** Book/Book chapter**	4	2.3
** Conference paper**	1	0.6
*** Total***	*174*	*100*
**Document Type**		
**Empirical research**	75	43.1
**Conceptual advancement**	38	21.8
**Position statement**	26	14.9
**Reports**	17	9.8
**Policy statement**	5	2.9
**Protocol**	2	1.1
**Scoping analysis**	1	0.6
**Others**	9	5.2
*** Total***	*174*	*100*

Most publications included case study – single or multiple – (*n* = 35; 20.1%) or qualitative research (*n* = 26; 14.9%). In several cases, mixed (quali-quantitative) methods (*n* = 15; 8.6%) or cross-sectional quantitative design (*n* = 10; 5.7%) were used. Some before-after studies (*n* = 3; 1.7%) or longitudinal analyses (*n* = 7; 4%) were retrieved. Systematic literature reviews, including scoping reviews (*n* = 5; 2.9%) and unsystematic overviews of the scholarly debate (*n* = 21; 12.1%) covered a small portion of reviewed items. Some articles developed a theoretical framework for engagement (*n* = 13; 7.5%). Many publications were opinion papers that appeared as commentaries (*n* = 26; 14.9%) or critical debates (*n* = 6; 3.4%; *e.g.*, through interviews with opinion leaders in parallel discussion of engagement issues in non-academical journals). The remaining publications were policy insights (*n* = 2; 1.1%), editorials (*n* = 2; 1.1%), letters to the editor (*n* = 1; 0.6%), and other contributions based on action research (*n* = 3; 1.7%) (Table 2).

**Table 2:** Study designs, methodologies, and approaches

Design/Methodology/Approach	n	%
**Case study**	35	20.1
**Commentary**	26	14.9
**Qualitative study**	25	14.4
**Review (not systematic)**	21	12.1
**Mixed quali/quantitative method**	15	8.6
**Theoretical development**	13	7.5
**Cross-sectional quantitative**	10	5.7
**Longitudinal**	7	4
**Critical debate**	6	3.4
**Systematic review or scoping review**	5	2.9
**Before-after study**	3	1.7
**Policy insight**	2	1.1
**Editorial**	2	1.1
**Letter**	1	0.6
**Others**	3	1.7
***Total***	*174*	*100*

### Conceptualizing physician engagement

#### Analysis of definitions in the scholarly debate

Full-text analysis demonstrates the relevant overlap between the concepts of physician engagement and clinician engagement. They are used mainly as synonyms, but with a specific focus on doctors in the former case and a broader consideration of the health care professional roles in the latter.

The primary focus of more than half items (56.3%; *n* = 98) was physician engagement, whilst the other (43.7%; *n* = 76) used the concept as a secondary or correlated theme for the study objective. The concept of physician/clinician engagement was clearly defined or explicitly reported in 23.6% (*n* = 41) papers, while five mentioned it as a correlated topic. We could not find consistent definitions of engagement. Drawing from the literature on psychological and personal conditions for job engagement [43], in many cases, the concept of engagement is related to “vigor, dedication, and absorption” in the workplace – the opposite of burn out – and is associated with a positive state of mind and sense of accomplishment and pride to contribute to organizational success [41, 44–49]. Consistent with this understanding, Owens et al. [50] argued that engagement entails a cognitive, emotional, and behavioral connection with the organization’s mission, vision, and values and, as such, it ensures that a discretionary effort is released so that they are prepared to “go the extra mile” for their organization [51]. Macinati et al. [52] defined managerial job engagement as “the harnessing of a medical manager’s full self in terms of cognitive and emotional energy in their managerial role performance”.

Other studies focused on the physicians’ involvement within their normal job roles in a health care organization and underlined its positive link with organizational performance and quality of care [38, 53–58]. Clinician engagement is defined as the active involvement of physicians in planning, delivering, improving, and assessing health services through the use of clinical skills, knowledge, and experience [59], also supporting organizational projects and decisions for enhancing [60]. Engagement implies the health professionals’ willingness to alter their behavior and involve themselves in processes of organizational change [61], taking an “active interest” in organizational excellence [62]. Some physicians may be actively interested in the quality of their workplace and motivated to assume a leadership role to improve it [16].

The Department of Health of the British National Health Service (NHS) (2013) defines engagement as “the mutual understanding and cooperation between different professions/cultures leading to joint working” (p. 8) between health professionals and financial managers to improve the quality of care while becoming more productive and efficient [63]. Lee and Cosgrove (2014) found that physician engagement is conventionally defined as the extent to which professionals see their future intertwined with that of the organization; however, engagement demands more than mere cooperation (*e.g.,* an agreement not to sabotage) and entails full collaboration for improvement [64].

In a reworking of previous concepts, broader definitions define clinician engagement as the degree to which physicians feel fulfilled and satisfied at work, supported within their organization, and motivated because they can suggest and implement ideas for improvement. Consequently, they are willing to recommend their organization as an excellent place to work or be treated [16, 65, 66]. Some concepts were simply general ideas about engagement or how physicians can be engaged [67–70]. As an illustrative example, it usually overlaps with leadership and is used as a synonym for leading capabilities [71–73]. Indeed, leadership is one of the distinguishing characteristics of clinician engagement.

#### Rationale for engagement

Many studies explicitly defined their rationale (*n* = 135, 77.6%). Occasionally, multiple rationales were undertaken in a single contribution. We identified seven rationales for engaging physicians:

1. Improving organizational performance (e.g., effectiveness, efficiency, costs reduction, *et similia*) (*n* = 52);
2. Sustaining a quality and safety culture throughout the organization (*n* = 50);
3. Nurturing service or process improvement and organizational change (*n* = 23);
4. Facilitating teamwork among health care professionals, between clinicians and managers, and between clinicians and administrative staff (*n* = 13);
5. Advancing job satisfaction and reducing burnout (*n* = 11);
6. Improving skills in decision-making, responsibility, and accountability (*n* = 11);
7. Promoting leadership and managerial skills (*n* = 9).

Table 3 presents the number of publications per rationale and year of publication. The first three categories address organizational issues, because engagement is seen as a means to achieve the mission of healthcare organizations, enhance their performance, and ensure continuous process improvement. Categories 4) and 5) address intra-organizational relationships between health care professionals [40]: greater engagement is linked to greater job satisfaction and less burnout, to closer collaboration among professionals, and to bridging the divide between physicians, managers and administrative staff [74]. The last two categories are related to the improvement of individual knowledge, skills, and attitudes.

**Table 3:** Rationales for physician engagement (*n* = 135)

Year	Organizational Performance	Quality & Safety	Service Improvement & Change Management	Teamwork	Decision-making & Accountability	Job Satisfaction	Leadership & Managerial Skills
**1993**		1					
**1997**		1			1		
**1998**			1				
**2001**		1					
**2005**	1						
**2007**		1	1				
**2008**		1			1		1
**2009**	1	4					
**2010**	1	3		1	2		2
**2011**	4	2	2		1		
**2012**	8	8	1		1		
**2013**	4	3	1	2			2
**2014**	6	4	5	2		1	
**2015**	5	4	2		2	1	2
**2016**	4	4	3	1	1	3	2
**2017**	9	4	3	2	1	1	
**2018**	3	3	1	2		1	
**2019**	3	3	1	2		1	
**2020**	3	3	2	1	1	3	
**TOTAL**	**52**	**50**	**23**	**13**	**11**	**11**	**9**

From a longitudinal perspective, physician engagement has been initially rooted in quality and safety improvement initiatives. In the early 21^st^ century, research began to explore other dimensions: the interplay between engagement and organizational performance and the relevance of engagement in service and process transformation. The main advantages of physician engagement were associated with improvement in the management of health care in response to patients’ needs, while preserving efficiency and sustainability, which are essential to retain institutional legitimacy towards stakeholders.

Several papers originated from the rationale to improve leadership and managerial skills of health care professionals, along with decision-making and accountability skills. This may be explained by the fact that they are considered means for physician engagement rather than its logical foundations. Lastly, greater interest in job satisfaction and teamwork echoed the wider acceptance of engagement as a psychological state in contrast to burn out.

### Features of physician engagement

#### Engagement practices and areas of involvement

Engagement can take different forms. We matched each publication with the most relevant form of engagement and found that multiple practices were often applied. Several studies examined formal types of involvement (Table 4), such as the appointment of health professionals to middle management roles (head of clinical directorates or specialty units; *n* = 9; 5.2%), membership of boards (*n* = 4; 2.3%), or assignment of top management positions (*e.g.,* chief medical officer [75]; *n* = 1; 0.6%). Some studies reported a link between chief executives with a medical background and organizational performance and between clinically qualified managers and organizational performance [19].

**Table 4:** Forms of engagement

Types	n	%
Involvement of frontline physicians in managerial positions and innovation projects	70	40.2
Leadership and soft skills and organizational roles	47	27
Physician engagement in organizational culture	20	11.5
Physicians appointed to middle management positions	9	5.2
Physician engagement in the assessment of organizational performance	7	4
Membership on organizational boards	4	2.3
Physician engagement in human resource management	4	2.3
Physicians appointed to managerial positions (CEO or medical director)	1	0.6
Other/unclear	12	6.9
*Total*	*174*	*100*

Most studies (*n* = 70; 40.2%) discussed the involvement of frontline professionals in managerial positions and innovation projects, focusing on quality and safety improvement projects, organizational change, and data management. Another critical research area concerned the role of leadership in building an informal work climate that fosters engagement (*n* = 47; 27%) for achieving organizational targets, such as quality improvement. Other papers discussed the role of clinician engagement in shaping the organizational culture for improving quality and innovation, as well as striking a balance between professional culture and managerial culture (*n* = 20; 11.5%), in assessing organizational performance (*n* = 7; 4%), and in managing people (*n* = 4; 2.3%). Increasing importance was attached to the physicians’ ability to use real-time dashboards displaying data metrics and appreciate how they affect clinical, financial, and operational issues in organizational performance [76]. Some studies were categorized as “other/unclear” (*n* = 12; 6.9%) since we were unable to pinpoint a specific form of engagement [63, 77–80] or it was not specified [41, 48, 81]. Several studies examined particular types of engagement that fell outside our categories, such as a novel program involving a physician quality officer [82] or the engagement of frontline physicians as supply chain managers [83, 84].

The main areas of engagement are linked to enhancing the delivery of health services, especially quality and safety (*n* = 54; 31%) and achieving organizational effectiveness (*n* = 29; 16.7%). Many studies focused on physicians’ involvement in organizational development processes by defining and implementing projects and innovations that impact diverse areas of health care management (*n* = 29; 16.7%). Other areas concerned the professional development of clinicians (*n* = 14;8%). Furthermore, several studies reported on how doctors can be involved in performance management (*n* = 14; 8%) or strategic decisions (*n* = 14; 8%). Ten items (5.7%) were focused on human resource management, motivation, assessment, and evaluation. Ten were categorized as other/unclear because the area of engagement was not specified [78, 85, 86] (Table 5).

**Table 5:** Areas of physician engagement

**Areas of engagement**	n	**%**
Health service quality & safety	54	31
Health service effectiveness	29	16.7
Organizational development	29	16.7
Professional development	14	8
Performance management	14	8
Strategy	14	8
Human resource management	10	5.7
Other/unclear	10	5.7
*Total*	*174*	*100*

#### Relationship between physician engagement and organizational performance

Most papers focused on clinician engagement for improving health care services and organizational performance. The four key dimensions were: 1) quality and safety; 2) patient outcomes and experience; 3) efficiency and costs; and 4) staff satisfaction. Physician engagement was primarily reported to impact on quality and safety improvement [15, 56, 67, 87–93]. Health care organizations increase their performance by soliciting the active participation of health professionals in quality and safety projects, and their engagement in the development and implementation of performance management activities for achieving better outcomes such as: data collection [94] and setting up standardized frameworks for benchmarking internal quality against external measures [95]. Moreover, the involvement of physicians in risk mitigation has been found to produce benefits in quality enhancement, leading to a reduction of malpractice and resource misuse [96].

Patients benefit from physician engagement due to more effective cross-boundary work [97]. Such advantages are triggered by timelier and safer care because of improved clinical practice [98]. Engaged health professionals can boost enhancement in organizational processes and dynamics, resulting in improved outcomes [67, 99, 100], better patient experience and satisfaction [49, 101, 102], and lower complication rates [103]. Many studies linked engagement with improved financial outcomes [49, 56, 96, 99], cost savings [100, 104], and benefits from reinvested funds [93]. Such evidence supports the implications of engagement on cost reduction and efficiency gain.

Physician engagement has been also found to generate a positive workplace atmosphere by fostering job satisfaction and commitment. This is expected to translate into greater work performance [102] and productivity [19, 73]. In fact, physician engagement has been associated with positive organizational outcomes (*e.g*., organizational commitment, job performance, less staff turnover) and individual outcomes, including better physical/psychosomatic health and proactive behaviors [46], determining lower rates of staff turnover and burnout [19, 49, 56, 73, 105, 106]. It may also improve the performance of health care teams via a greater commitment and collaboration in the workplace [107].

Owens et al. (2017) found that U.S. organizations in the top quartile for strength of organizational culture outperform those in the bottom quartile for every physician engagement domain (including hospital efficiency, hospital quality, overall satisfaction) [50]. The top hospitals outperformed those in the bottom quartile three to four times in most domains (except for admission and discharge procedures and medical records and clinical information*,* which are linked to operations management rather than to outcomes).

### Methods and scales for evaluating physician engagement

Methods and tools for measuring physician engagement in health care organizations were described in 21 studies. A widely used instrument is the Medical Engagement Scale (MES), developed in the UK by the NHS Institute for Innovation and Improvement and the Academy of Medical Royal College as part of the Enhancing Engagement in Medical Leadership project [19, 54, 73]. The scale has been revised in years of testing in numerous NHS trusts involving thousands of physicians. Appreciated as a reliable and valid measure of physician engagement, it is quick and relatively easy to administer and complete [16]. It is published as either an 18-item or a 30-item tool. The 18-item version of the scale measures engagement on three dimensions (meta-scales): 1) feeling valued and empowered; 2) having purpose and direction; and 3) working in an open culture. The 30-item version includes additional subscales, including: meta-scale 1 investigates climate for positive learning and good interpersonal relationships, while meta-scale 2 investigates appraisal and rewards; and participation in decision-making and change; meta-scale 3 investigates development orientation; and commitment and work satisfaction. The MES has been used to assess the link between engagement and performance [19, 53]. To date, it has not been tested outside the UK and many of the items may not be appropriate for other country contexts and health care systems.

Two documents in the grey literature described a self-assessment tool [63] and a medical engagement checklist [108] that support organizations and/or individuals in medical leadership roles. The idea was to have a practical instrument rather than a scientific tool to assess how engagement is sought and developed. Other assessment questionnaires focused on the levels, determinants, and barriers to engagement, such as the hospital-physician engagement agreement [109] or the systems approach to patient safety and quality [110].

Dellve et al. [46] devised an instrument for assessing clinician engagement in organizational redesign by drawing on insights from a qualitative study by Lindgren et al. [111]: central statements and substantive codes related to positive and negative attitudes, beliefs, and motivation for engaging in organizational improvements were articulated into items with a 4-point response scale, piloted with health care clinicians to determine item clarity and construct validity. Other tools included the Swedish Scale for Work Engagement and Burnout (SWEBO) and two scales to assess engagement in patient safety and quality of care. In their qualitative study based on semi-structured interviews, Taitz and colleagues [20] assessed themes common to organizations with significant physician involvement to explore how organizations engage professionals in quality-and-safety improvement activities. The study’s main aim was to identify the key facilitators, barriers, and costs of physician engagement based on a 20-item questionnaire. Spaulding et al. [112] asked 38 leaders at a large, metropolitan multi-hospital health system to define critical success factors in their physician engagement initiative. The perceptions of leadership qualities were categorized into four broad themes: 1) relationship and communication; 2) providing positive experience; 3) integration; and 4) accountability and quality.

Drawing on the definition of physician engagement by the UK NHS as “the degree to which an employee is satisfied in their work, motivated to perform well, able to suggest and implement ideas for improvement, and their willingness to act as an advocate for their organization by recommending it as a place to work or be treated”, Rinne et al. [65] devised a questionnaire with two dichotomous items and four open-ended questions that investigated the opinions of U.S. hospital administrators on the perceived determinants of engagement and the barriers to health professionals engagement. Keller et al. [66] recruited 20 physicians from diverse specialties and 20 health care administrators for semi-structured interviews to determine whether cultural differences could affect physician engagement at the institutional and the organizational level. Since the authors developed these assessment methods and tools according to the study aims, they cannot be considered reliable and replicable.

Ireri and colleagues [113] administered a questionnaire based on the 80 competency outcomes of the Medical Leadership Competency Framework [114] and found an overlap between engagement theories and leadership theory. In their study, Kreindler et al. [61] use the social identity approach (SIA), comprising personal identity and social identity to evaluate physician engagement strategies on a continuum from individualism to a shared identity.

Several scales from other scientific fields and industries have been used in the reviewed items, though not developed specifically for health care organizations. Examples are the 12-item Gallup Q12 survey instrument [115] and the Utrecht Work Engagement Scale (UWES), a 9-item self-report questionnaire consisting of three subscales (“vigor”, “dedication”, “absorption”) [106, 116]. Other studies administered national surveys or proxies for engagement, especially in the US. Owens and colleagues (2017) used HealthStream surveys to create nationally representative databases and statistically validated surveys for benchmarking physician engagement [50]. In their longitudinal study, Scher et al. [48] used the Advisory Board Survey Solution (ABSS), with a dataset of over 55,000 physicians who expected to remain in their own organization (avoiding bias of those expecting to leave) and scored the responses to four categories of items: “engaged” was linked to the category of “doctors highly loyal and committed to the organization”; “not engaged” was related to the categories “content” (satisfied but no extra effort to help the organization succeed), “ambivalent” (not invested in the organization), and “disengaged” (actively unhappy with the organization). Other proxy indicators for engagement were first-year turnover, sick time utilization, and workplace injuries or quality-related metrics like hand-hygiene compliance [99].

### Enablers and barriers of physician engagement

About 60% of reviewed items (*n* = 104; 59.8%) discussed factors impeding physician engagement. The main issues concern individual attitudes and skills, conflict between managerial and clinical culture [56] mistrust toward managers [57, 117–119], and frustration from a sense of loss of autonomy [20, 110, 118] Due to these factors, physicians are reluctant to take on a management role [46, 120, 121].

Specialists (*e.g.*, surgeons) are less subject to managerial control because of their organizational cultures, being traditionally individualistic and adverse to competition and rationalization [67, 122]. According to Bohmer (2012, p.26), “yet frontline doctors are unprepared and unschooled for a leadership role, often unsupported in this work” [123]. Malby et al. [86] identified sources of tension that hinder engagement:

1. a perception of leadership based on personal (credibility, respect, trust) and expert power (knowledge of clinical conditions) positional power;
2. a focus on professionalization (knowledge, personal accountability, unilateral autonomy, decision-making) rather than on professionalism (reflection, interdependent decision-making, collective responsibility);
3. illusion of expertise and evidence.

Conversely, soft skills are crucial factors for engagement, such as communication abilities (listen and act upon the informed judgement of others), political dexterity (convey reasoned and rational arguments clearly), clinical credibility (gain respect by peers), personality, behaviors, and moral values [124].

In the report by Metrics@Work Inc., Grimes, and Swettenham (2012) systematized the most relevant drivers of physician engagement, grouped into five categories [125]: 1) management and leadership (*e.g.*, governance, decision-making, communication, culture, mission, vision, values, organization and delivery of care and services, human resource management, individual relationships, and personal character); 2) funding and financing (*e.g.,* payment systems, rewards, recognition, incentives); 3) quality initiatives (*e.g.*, quality monitoring and improvement, metrics, standardization); 4) regulation, legislation, liability (*e.g.,* self-regulation, accountability, credentialling, bylaws, codes of ethics, competencies); 5) information and communication technologies (*e.g.,* electronic medical records, innovation, privacy, consent).

Leadership skills are among the most powerful tools to foster physician engagement. Hence, a lack of leadership attitudes, persuasion techniques, mentoring, conflict management, and coaching can all hinder involvement [30]. Conversely, factors facilitating engagement may be a future-focused and outward-looking culture, increased attention to recruitment and selection of doctors to be trained for leadership and management, development of leadership opportunities, and provision of support and effective communication [53]. Other barriers to engagement are the lack of managerial and technical skills and experience [126, 127], limited understanding of health care systems and management jargon [30] inadequate financial and accounting management skills [30, 56] and quality-improvement skills in specific projects [20].

Studies signal the need to expand the skill set for engagement (Table 6). Technical and managerial competencies are crucial (*n* = 57; 32.8%): physicians should master management methods and techniques (budget, quality, and safety improvement) and develop a toolbox for use when involved in decision-making processes, organizational development initiatives, quality improvement, *et similia*. Interpersonal and relational skills (*n* = 43; 24.7%) are fundamental to promote engagement among professionals and between clinicians and managers. They comprise the ability to create conditions for teamworking, motivate people, and solve and minimize conflicts in decision-making and during the implementation of organizational projects. Personal soft skills are essential for active involvement (*n* = 33; 19%): they comprise a strong work ethic, integrity, flexibility, and adaptability to change management and improvement projects. Conceptual and strategy skills (*n* = 19; 10.9%) were mainly related to the ability to examine the external and the internal environment, grasp the whole picture, set priorities, and define plans or select strategic choices. The final category (*n* = 22; 12.6%) listed in Table 6 comprises 11 studies that reported a bundle of skills without explaining which was the most important [19, 53, 54, 63, 70, 75, 109, 111, 114, 128, 129], and 11 studies in which the critical skills for engagement were either unclear (*n* = 2) or not specified (*n* = 9). Summarizing, improving physicians’ attitudes may increase their overall work satisfaction and promote engagement [120].

**Table 6:** Skills for physician engagement

**Skills for improving engagement**	n	**%**
Technical/managerial skills	57	32.8
Interpersonal and relational skills (conflict management & teamworking)	43	24.7
Personal soft skills	33	19
Conceptual/strategy skills	19	10.9
Other/unclear	22	12.6
*Total*	*174*	*100*

Opdahl Mo [130] reported that engagement is time-consuming and time constraints are significant obstacles to involving physicians in organizational dynamics [117, 131–134]. From a management perspective, other hindering factors are inadequate resources and competing tasks, lack of information-system support and/or trust in data, lack of training, inadequate rewards (financial and non-financial), staff turnover, disinterest, and the wish to maintain professional autonomy [74, 101, 133, 135, 136]. Failure may result from poor communication and inter-professional relationships [132] or lack of support from general managers and hesitation to deal with complex issues [77]. Dickinson and colleagues [77] found evidence that doctors considered the lack of clear career structures in management roles and financial incentives as barriers to engagement.

Local staffing constraints, lack of resources, competing demands, and changes in organizational governance and priorities challenge implementing quality improvement projects [137, 138]. The lack of a shared focus on quality improvement and limited engagement in management were the main reasons for negative attitudes toward clinical governance [139]. Finally, skepticism may stem from the perception that physician involvement is not translated into fundamental changes in the delivery of services and patient care, but instead adds to an already heavy workload without gain [136].

Bickell and colleagues [117] identified four factors that discourage engagement in quality improvement projects: 1) few institutions are willing to involve the entire physician staff in management decisions; 2) if managed by a top-down approach, it is difficult to differentiate these projects from bureaucratic and punitive quality assurance activities; 3) many physicians believe that most projects are cost-control activities masquerading as a quality improvement; and 4) lack of scientific rigor in studies and immediate evidence of improved outcomes.

From this standpoint, health care organizations can play a critical role in improving engagement (Table 7), which can be direct (*e.g.*, supporting physicians’ involvement) or indirect (*e.g.*, enabling skills and competencies for engagement). There are policies for creating a work environment that actively supports involvement (*n* = 50; 28.7%), as well as training and coaching sessions helping to develop skills and attitudes useful for engagement (*n* = 40; 23%).

**Table 7:** Policies for engagement

**Organizational policies for stimulating engagement**	n	**%**
Supporting engagement	50	28.7
Training and coaching	40	23
Empowering/enabling physicians	33	19
Communication to professionals	20	11.5
Incentives/awards	13	7.5
Other/unclear	18	10.3
Total	174	100

Empowering and enabling physicians is another policy (*n* = 33; 13) fostering their participation in decision-making, problem-solving, and goal-setting, while increasing their responsibility and accountability. Fostering a climate where clear and continuous communication between senior management and physicians is vital to develop a positive attitude toward the organization (*n* = 20; 11.5%). Finally, extrinsic rewards, such as incentives and awards, are powerful means to support engagement (*n* = 13; 7.5%). The final category (others/unclear) comprises studies (*n* = 12) that report on organizational policies for improving engagement [47, 57, 58, 60, 63, 80, 111, 125, 140–142].

All high-performing organizations have a demonstrable commitment to quality improvement. Furthermore, better quality is delivered by organizations where the physician staff is cohesive and structured to support interaction with senior leadership [89]. Collaboration between executive and clinical directors can help to improve communication and knowledge [69]. Quality improvement interventions should aim to align staff at multiple levels in the organization: a commitment by senior and middle managers to quality improvement is crucial to foster the engagement of front-line clinicians [143].

The cultural context, the technical support, the ability to communicate clear strategies and goals, and the organizational structure can all shape physicians’ attitudes to clinical governance and, in turn, prompt their engagement and the success of quality improvement initiatives [139]. Identification of physician champions and a culture oriented toward innovation can foster engagement by empowering health professionals to achieve the expected results of management decisions [60]. Kaissi developed a three-tiered integrative framework that managers should implement [16]:

1. Create clear and efficient communication channels through a physician communication plan;
2. Build trust, understanding, and respect by involving physicians in strategy-making;
3. Identify and develop physician leaders to help engage other physicians by creating new structures and roles for physician leaders.

## Discussion

This scoping review enabled us to deliver an overview of the extant scholarly debate about physician engagement, highlighting its contribution to advancing the functioning of health care organizations. There is a consolidated debate on physician engagement. However, there is no consensus on its conceptualization. Two dominant perspectives have been spotted. Part of the literature conceives engagement as a positive state of mind characterized by physicians’ cognitive and affective participation in organizational dynamics and unleashing vigor and dedication to work. Other contributions frame engagement as formal and informal attempts to empower health professionals, encouraging them to partake in management decisions and steer health care organizations. Despite the apparent differences, these two definitions are not at odds. Engagement relies on a motivational state, which enhances physicians’ commitment to advancing organizational performance. This positive motivational state is boosted by management practices facilitating physicians’ participation in setting the agenda of health care organization. Hence, engagement can be understood as a multifaceted construct consisting of a psychological commitment that spurs a sense of belongingness to the organization and the opportunity to participate in decision-making.

Evidence of this twofold interpretation can be found in the rationales for physicians’ engagement retrieved in the reviewed contributions. Soft and hard rationales lay behind the decision to involve physicians in organizational dynamics. The soft rationale implies nurturing the physicians’ satisfaction with their work conditions. Engagement fosters a positive work environment where the individual contribution to organizational performance is recognized, and incentives exist to impact organizational processes. By reducing negative work attitudes and strengthening organizational commitment, energy can be devoted to dealing with organizational problems, with positive implications for value generation. The hard rationale is consistent with the conceptualization of engagement as a management practice to achieve corporate democracy and participation. Engagement raises the health professionals’ awareness of the complexity of decision-making, appreciating the need to pursue both service quality and organizational efficiency. Such awareness supports physicians in learning how to conceive and drive change management processes that improve the health care institutions’ viability.

Literature has discussed different practices conducive to physician engagement. The articulation of such practices follows the discrimination among hard and soft factors adopted to delve into the rationales of engagement. Many papers have attached emphasis to informal factors, pointing out that leadership represents the first step for achieving physicians’ engagement in management decisions. Moreover, reconfiguring the organizational culture in a perspective of openness and empowerment is essential to foster engagement. In fact, a hierarchical culture established on stability and predictability prevents health care organizations from taking advantage of engagement. As far as hard factors are concerned, different degrees of engagement can be identified, such as the physicians’ participation in co-assessing organizational performance, their involvement in management duties, the establishment of executive boards, and the creation of task forces and committees to facilitate the empowerment of health professionals. The literature consistently maintains that formal and informal practices to achieve engagement should be aligned to fully engage physicians in coping with organizational challenges.

The extant scholarly debate pinpointed two main areas in which physicians’ involvement in steering health care organizations can be implemented. First, engagement is predominantly applied to the achievement of health services’ effectiveness, empowering physicians to partake in redesigning organizational processes to improve safety, quality, and appropriateness of care. Second, engagement is usually targeted to get structural improvements, enhancing the context within which health services are delivered, and professional gains, advancing physicians’ self-determination and ability to impact organizational performance. Alongside these two areas, the scholarly debate devotes growing attention to physicians’ participation in shaping strategic management decisions, performance management issues, and people management.

This scoping review highlighted a relatively under-researched topic concerning measuring physicians’ engagement. Although different attempts have been realized, scholars and practitioners do not agree on the tools to obtain a consistent and dependable measurement of engagement. Extant measures show three main shortcomings: 1) they are affected by content ambiguity; 2) they are applied to specific categories of health professionals, preventing us from obtaining comparative insights; and 3) they suffer from an institutional bias, being predominantly contextualized in the Anglo-Saxon setting. The lack of dependable tools to measure physicians’ engagement prevents us from making sense of its micro, meso, and macro organizational implications.

Last, the debate has focused on the skills and competencies required to assist physicians in achieving engagement. Interestingly, soft skills, including conflict management, teamworking, and relational abilities, have been identified as key factors leading to successful engagement. From this standpoint, organizational policies to promote engagement should focus not only on creating a work environment that supports participation, but also on enhancing soft skills, entailing interventions aimed at empowering physicians and tailored actions directed at soliciting organizational citizenship behaviors. Such initiatives should be established on material incentives to support engagement, including rewards for individual and collective participation in decision-making processes and training sessions to raise awareness of relevant management challenges.

## Limitations

Despite the solid and replicable study protocol used in this research, several limitations affected the findings. We did not use a bibliometric approach to differentiate research streams in the scholarly debate, which could reveal consistencies and inconsistencies across scientific contributions. Moreover, we did not assess the quality and robustness of reviewed studies. Our study failed to provide a meta-synthesis of evidence in the current literature, which could have identified the determinants and implications of physician engagement. This review did not investigate the engagement of other nonphysician clinicians, such as nurses and general practitioners. This narrowed down the study breadth. Just as the values and interests of physicians in health care organizations may differ from those of other health professionals [144], so, too, their motivation or incentive to engage may differ in relative importance. Therefore, that the present review focused on physicians is warranted by its objective to gain a clear vision of the characteristics of engagement by such professionals. Lastly, yet importantly, this review included publications as of December 2020. This was done to avert potential bias in interpreting the study results. Previous studies emphasized organizational stress and health care staff burnout due to the COVID-19 emergency [145, 146]. Research after that date might have misrepresented the actual characteristics of engagement in non-emergency situations. As an aside, an empirical study on engagement in a private healthcare group in Thailand demonstrated that the COVID-19 pandemic did not influence the understanding and drivers of engagement, although the pandemic was found to increase the health professionals’ willingness to be engaged [147].

## Conclusion, implications, and avenues for further development

This scoping review presents current knowledge about physician engagement, illuminating its drivers. Scholars have crafted a multiplicity of approaches to define physician engagement and shed light on its implications. We noted a growing interest in the topic as it has evolved over the past 15 years in response to the pressure on health systems. Studies indicate that health care organizations need to promote engagement if they want to sustain innovation, meaningful work, and commitment. Further research is required to understand better how health care organizations can leverage engagement to enhance their viability. One area of focus concerns the determinants of engagement, including the factors that empower health professionals to achieve salience and relevance in the organization. Another area to be elucidated entails the professional attributes that prompt physicians’ involvement. Finally, the knowledge, skills, and attitudes that enable engagement must be carefully delineated. Future studies should inform organizational policies and management practices designed to empower physicians and enable them to partake in corporate decision-making. A socio-technical perspective [148] is helpful for this purpose, envisioning how soft organizational practices can be aligned with hard management interventions to pave the way for physicians engagement. Additional research should be directed at assessing the short and long-term implications of engagement. *Inter alia*, the short-term effects on work climate and job satisfaction and the long-term effects on organizational performance and excellence should be investigated. A simple tool to measure the level of engagement in health care organizations is required to operationalize the involvement of physicians in steering organizational dynamics and making management decisions. Finally, the COVID-19 pandemic may have changed the contents and forms of engagement in decision-making and organizational practices. Further studies are necessitated to shed light on how physician engagement will evolve in the post Covid-19 era, adding to what we currently know about engagement, resilience, and viability of modern health care systems.

### Supplementary Information

**Additional file 1: Appendix 1.** The list of items included in scoping review by year of publication (*n*=174).

**Additional file 2:** PRISMA-ScR checklist.

## Data Availability

The dataset of this study is available with the Supplementary Materials.
