# Supplementary material for: Exploring physician engagement in health care organizations: a scoping review
Source: BMC Health Serv Res. 2023 Sep 26;23:1029. doi: 10.1186/s12913-023-09935-1 (PMC10521513; doi:10.1186/s12913-023-09935-1)
Supplement: Supplementary file 1 — Additional file 1: Appendix 1. The list of items included in scoping review by year of publication (n=174). [file 12913_2023_9935_MOESM1_ESM.docx]

Appendix 1. The list of items included in scoping review by year of publication (n=174)

| **ID** | **Authors** | **Title** | **Publication Year** | **Document category** | **Source title** | **Document type** | **Design/methodology/approach** |
| --- | --- | --- | --- | --- | --- | --- | --- |
| 1 | Gates PE. | Clinical Quality Improvement: Getting Physicians Involved | 1993 | Article | Quality Review Bulletin | Empirical research | Case study/ies |
| 2 | Buchanan D, Jordan S, Preston D, Smith A. | Doctor in the process: the engagement of clinical directors in hospital management | 1997 | Article | Journal of Management in Medicine | Empirical research | Case study/ies |
| 3 | Weiner BJ, Shortell SM, Alexander J. | Promoting Clinical Involvement in Hospital Quality Improvement Efforts: The Effects of Top Management, Board, and Physician Leadership | 1997 | Article | Health Services Research | Empirical research | Cross-sectional quantitative study |
| 4 | Bickell NA, Aufses AH, Jr., Chassin M. | Engaging clinicians in a quality improvement strategy for early-stage breast cancer treatment. | 1998 | Article | Quality Management in Health Care | Empirical research | Case study/ies |
| 5 | Willcocks S. | Clinical Management and Cultural Diversity: The Cultural Context of Doctor Involvement in the Managerial Process | 1999 | Article | Health Services Management Research | Empirical research | Case study/ies |
| 6 | Lindenfeld S, Vlchek D. | Engaging physicians in continuous quality improvement | 2001 | Article | Advances in Renal Replacement Therapy | Position Paper | Theoretical development |
| 7 | Young AE. | The medical manager: a practical guide for clinicians | 2003 | Book | BMJ Publishing Group | Position Paper | Theoretical development |
| 8 | Guthrie M. | Engaging physicians in performance improvement | 2005 | Perspectives and others | American Journal of Medical Quality | Conceptual Paper | Commentary |
| 9 | Royal College of Physicians' iLab | Engaging clinicians in improving data quality in the NHS | 2006 | Report | Royal College of Physicians | Policy document | Other: Action Research |
| 10 | Puri AK, Bhaloo T, Kirshin T, Mithani A. | A comprehensive approach to effectively engage physicians during a hospital closure: Using the physician engagement model | 2006 | Article | Healthcare Management Forum | Empirical research | Case study/ies |
| 11 | Woodard F. | How to achieve effective clinical engagement and leadership when working across organisational boundaries: practical recommendations | 2007 | Report | Modernisation Initiative (web access: Mental Health Partnership) | Policy document | Policy Insight |
| 12 | Brand C, Ibrahim J, Bain C, Jones C, King B. | Engineering a safe landing: engaging medical practitioners in a systems approach to patient safety. | 2007 | Article | Internal Medicine Journal | Empirical research | Cross-sectional quantitative study |
| 13 | Hogan H, Basnett I, McKee M. | Consultants' attitudes to clinical governance : Barriers and incentives to engagement. | 2007 | Article | Public Health | Empirical research | Case study/ies |
| 14 | Harmond K. | Clinical engagement: so why should you even bother? | 2007 | Article | British Journal of Healthcare Management | Empirical research | Case study/ies |
| 15 | Neale G, Vincent C, Darzi AS. | The problem of engaging hospital doctors in promoting safety and quality in clinical care | 2007 | Article | Journal of The Royal Society for the Promotion of Health | Conceptual Paper | Review (not systematic) |
| 16 | Clark J, Armit K. | Attainment of competency in management and leadership: No longer an optional extra for doctors | 2008 | Article | Clinical Governance: An International Journal | Conceptual Paper | Qualitative study |
| 17 | Konteh F, Mannion R, Davies H. | Clinical governance views on culture and quality improvement | 2008 | Article | Clinical Governance: An International Journal | Empirical research | Cross-sectional quantitative study |
| 18 | Opdahl Mo T. | Doctors as managers: Moving towards general management? The case of unitary management reform in Norwegian hospitals | 2008 | Article | Journal of Health Organization and Management | Empirical research | Case study/ies |
| 19 | Spurgeon P, Barwell F, Mazelan PM. | Developing a medical engagement scale (MES). | 2008 | Article | International Journal of Clinical Leadership | Conceptual Paper | Theoretical development |
| 20 | Caverzagie KJ, Bernabeo EC, Reddy SG, Holmboe ES. | The role of physician engagement on the impact of the hospital-based practice improvement module (PIM) | 2009 | Article | Journal of Hospital Medicine | Empirical research | Qualitative study |
| 21 | Heenan M, Higgins D. | Engaging physician leaders in performance measurement and quality. | 2009 | Article | Healthcare Quarterly | Report | Case study/ies |
| 22 | Siriwardena AN. | Engaging clinicians in quality improvement initiatives: art or science? | 2009 | Perspectives and others | Quality in Primary Care | Position Paper | Editorial |
| 23 | Walsh KE, Ettinger WH, Klugman RA. | Physician quality officer: A new model for engaging physicians in quality improvement. | 2009 | Article | American Journal of Medical Quality | Report | Case study/ies |
| 24 | McKimm J, Rankin D, Poole P, Swanwick T, Barrow M. | Developing medical leadership: a comparative review of approaches in the UK and New Zealand | 2009 | Article | International Journal of Leadership in Public Services | Report | Review (not systematic) |
| 25 | Irish J. | Clinical Engagement for performance improvements | 2009 | Perspectives and others | Healthcare Quarterly | Position Paper | Commentary |
| 26 | NHS Institute for Innovation and Improvement & Academy of Medical Royal Colleges | Medical Leadership Competency Framework: Enhancing Engagement in Medical Leadership | 2010 | Report | NHS Institute for Innovation and Improvement & Academy of Medical Royal Colleges | Position Paper | Theoretical development |
| 27 | Clark J, Armit K. | Leadership competency for doctors: a framework. | 2010 | Article | Leadership in Health Services | Conceptual Paper | Review (not systematic) |
| 28 | Clarke ALL, Shearer W, Mcmillan AJ, Ireland PD. | Investigating apparent variation in quality of care: the critical role of clinician engagement | 2010 | Article | The Medical Journal of Australia | Report | Mixed, quali/quantitative methods |
| 29 | Coutts J. | Engaging physicians to improve quality. | 2010 | Article | Healthcare Quarterly | Report | Critical debate |
| 30 | Hockey PM, Bates DW. | Physicians' identification of factors associated with quality in high- and low-performing hospitals | 2010 | Article | Joint Commission Journal on Quality and Patient Safety | Empirical research | Qualitative study |
| 31 | Hayes C, Yousefi V, Wallington T, Ginzburg | A Case Study of Physician Leaders in Quality and Patient Safety, and the Development of a Physician Leadership Network | 2010 | Article | Healthcare Quarterly | Empirical research | Qualitative study |
| 32 | Parand A, Burnett S, Benn J, Iskander S, Pinto A, Vincent C. | Medical engagement in organisation-wide safety and quality-improvement programmes: Experience in the UK Safer Patients Initiative | 2010 | Article | Quality and Safety in Health Care | Empirical research | Qualitative study |
| 33 | Weller J, Thwaites J, Bhoopatkar H, Hazell W. | Are doctors team players, and do they need to be? | 2010 | Article | The New Zealand Medical Journal | Conceptual Paper | Review (not systematic) |
| 34 | Tinline G, Crowe K. | Improving employee engagement and wellbeing in an NHS trust | 2010 | Article | Strategic HR Review | Empirical research | Case study/ies |
| 35 | Atkinson S, Spurgeon P, Clark J | Engaging Doctors: What can we learn from trusts with high levels of medical engagement? | 2011 | Report | NHS Institute for Innovation and Improvement & Academy of Medical Royal Colleges | Empirical research | Case study/ies |
| 36 | Stoll L, Swanwick T, Foster-Turner J., Moss F. | Leadership development for junior doctors: What can we learn from “Darzi” Fellowships in clinical leadership? | 2011 | Article | International Journal of Leadership in Public Services | Empirical research | Mixed, quali/quantitative methods |
| 37 | Baker GR, Denis JL. | Medical leadership in health care systems: from professional authority to organizational leadership | 2011 | Article | Public Money & Management | Conceptual Paper | Theoretical development |
| 38 | Cammisa C, Partridge G, Ardans C, Buehrer K, Chapman B, Beckman H. | Engaging Physicians in Change: Results of a Safety Net Quality Improvement Program to Reduce Overuse | 2011 | Article | American Journal of Medical Quality | Empirical research | Before-after study |
| 39 | Dobrow M, Neeson J, Sullivan T. | Canadian Chief Executive Officers' Prescription for Higher Quality: More Clinical Engagement, Shared Accountability and Capacity Development | 2011 | Article | Healthcare Quarterly | Empirical research | Cross-sectional quantitative study |
| 40 | Garg S, van Niekerk J, Campbell M. | Medical leadership: Competencies in action. | 2011 | Article | Advances in Psychiatric Treatment | Conceptual Paper | Review (not systematic) |
| 41 | Ireri S, Walshe K, Benson L, Mwanthi MA. | A qualitative and quantitative study of medical leadership and management: experiences, competencies, and development needs of doctor managers in the United Kingdom. | 2011 | Article | Journal of Management & Marketing in Healthcare | Empirical research | Mixed, quali/quantitative methods |
| 42 | Malby R, Edmonstone J, Ross D, Wolfenden N. | Clinical leadership: the challenge of making the most of doctors in management. | 2011 | Article | British Journal of Hospital Medicine | Conceptual Paper | Commentary |
| 43 | Pham HH, Bernabeo EC, Chesluk BJ, Holmboe ES. | The roles of practice systems and individual effort in quality performance. | 2011 | Article | BMJ Quality and Safety | Empirical research | Qualitative study |
| 44 | Snell AJ, Briscoe D, Dickson G. | From the inside out: The engagement of physicians as leaders in health care settings | 2011 | Article | Qualitative Health Research | Empirical research | Qualitative study |
| 45 | Spurgeon P, Mazelan PM, Barwell F. | Medical engagement: A crucial underpinning to organizational performance | 2011 | Article | Health Services Management Research | Empirical research | Cross-sectional quantitative study |
| 46 | Swanwick T, McKimm J. | What is clinical leadership...and why is it important? | 2011 | Article | The Clinical Teacher | Conceptual Paper | Review (not systematic) |
| 47 | Williamson S. | Engagement between medical staff and managers | 2011 | Article | British Journal of Healthcare Management | Conceptual Paper | Commentary |
| 48 | British Medical Association (BMA) | Doctors' perspective on Clinical Leadership | 2012 | Report | Health Policy & Economic Research Unit - BMA | Policy document | Qualitative study |
| 49 | Bohmer R. | The instrumental value of medical leadership: Engaging doctors in improving services | 2012 | Report | The King's Fund | Position Paper | Theoretical development |
| 50 | Clark J. | Medical engagement: Too important to be left to chance | 2012 | Report | The King's Fund | Conceptual Paper | Review (not systematic) |
| 51 | Bonias D, Leggat SG, Bartram T. | Encouraging participation in health system reform: Is clinical engagement a useful concept for policy and management? | 2012 | Article | Australian Health Review | Conceptual Paper | Systematic review |
| 52 | Busari JO. | Management and leadership development in healthcare and the challenges facing physician managers in clinical practice | 2012 | Perspectives and others | International Journal of Clinical Leadership | Position Paper | Case study/ies |
| 53 | Carruthers C, Levinson W. | Engaging clinicians through intrinsic incentives. | 2012 | Perspectives and others | Healthcare Quarterly | Other | Critical debate |
| 54 | Dwyer AJ, Becker G, Hawkins C, McKenzie L, Wells M. | Engaging medical staff in clinical governance: Introducing new technologies and clinical practice into public hospitals | 2012 | Article | Australian Health Review | Empirical research | Case study/ies |
| 55 | Healey C, Fearnley D, Chivers M, Wadoo O, Kinderman P. | Mixed-methods evaluation of a leadership development programme for higher specialist trainees | 2012 | Article | Psychiatrist | Empirical research | Mixed, quali/quantitative methods |
| 56 | Greening J. | How can we improve the effective engagement of doctors in clinical leadership?: The view of a consultant psychiatrist | 2012 | Article | Leadership in Health Services | Position Paper | Review (not systematic) |
| 57 | Clark J. | Medical leadership and engagement: no longer an optional extra | 2012 | Article | Journal of Health, Organisation and Management | Conceptual Paper | Review (not systematic) |
| 58 | Sawka C, Ross J, Srigley J, Irish J. | The crucial role of clinician engagement in system-wide quality improvement: the Cancer Care Ontario experience | 2012 | Article | Healthcare Quarterly | Report | Case study/ies |
| 59 | Scott CG, Theriault A, McGuire S, Samson A, Clement C, Worthington JR. | Developing a physician engagement agreement at The Ottawa Hospital: a collaborative approach. | 2012 | Article | Healthcare Quarterly | Empirical research | Case study/ies |
| 60 | Taitz JM, Lee TH, Sequist TD. | A framework for engaging physicians in quality and safety | 2012 | Article | BMJ Quality and Safety | Empirical research | Qualitative study |
| 61 | Thorp J, Baqai W, Witters D, Harter J, Agrawal S, Kanitkar K, Pappas J. | Workplace Engagement and Workers’ Compensation Claims as Predictors for Patient Safety Culture | 2012 | Article | Journal of Patient Safety | Empirical research | Longitudinal analysis |
| 62 | Rowling E. (Ed.) | Leadership and engagement for improvement in the NHS: Together we can | 2012 | Report | The King’s Fund | Conceptual Paper | Review (not systematic) |
| 63 | Kaissi A. | A Roadmap for Trust: Enhancing Physician Engagement | 2012 | Report | Canadian Policy Network & Regina Qu'Appelle Health Region | Position Paper | Different study designs |
| 64 | [Metrics@Work Inc., Grimes K, Swettenham J.](mailto:Metrics@Work%20Inc.,%20Grimes%20K,%20Swettenham%20J.) | Compass for Transformation: Barriers and Facilitators to Physician Engagement | 2012 | Report | Canadian Policy Network & Regina Qu'Appelle Health Region | Position Paper | Different study designs |
| 65 | Dickson G. | Anchoring Physician Engagement in Vision and Values: Principles and Framework | 2012 | Report | Canadian Policy Network & Regina Qu'Appelle Health Region | Position Paper | Review (not systematic) |
| 66 | Akosa AN. | Physician Engagement is Critical to the Success of any Accountable Care Organization | 2013 | Article | Journal of Managed Care Medicine | Report | Mixed, quali/quantitative methods |
| 67 | Bååthe F, Norbäck LE. | Engaging physicians in organisational improvement work | 2013 | Article | Journal of Health, Organisation and Management | Empirical research | Qualitative study |
| 68 | Bismark MM, Walter SJ, Studdert DM. | The role of boards in clinical governance: activities and attitudes among members of public health service boards in Victoria | 2013 | Article | Australian Health Review | Empirical research | Cross-sectional quantitative study |
| 69 | Dickinson H, Ham C, Snelling I, Spurgeon P. | Medical leadership arrangements in English healthcare organisations: Findings from a national survey and case studies of NHS trusts | 2013 | Article | Health Services Management Research | Empirical research | Mixed, quali/quantitative methods |
| 70 | Edwards MT. | A Longitudinal Study of Clinical Peer Review's Impact on Quality and Safety in U.S. Hospitals | 2013 | Article | Journal of Healthcare Management | Empirical research | Longitudinal analysis |
| 71 | Lindgren Å, Bååthe F, Dellve L. | Why risk professional fulfilment: a grounded theory of physician engagement in healthcare development | 2013 | Article | The International Journal of Health Planning and Management | Empirical research | Qualitative study |
| 72 | McWilliams C, Manochin MM. | Engaging junior doctors: Evidence from "open spaces" in England. | 2013 | Article | Journal of Health, Organisation and Management | Empirical research | Qualitative study |
| 73 | Patel B. | Engaging busy physicians key to improving outcomes | 2013 | Perspectives and others | Managed Care | Other | Commentary |
| 74 | Ravaghi H, Heidarpour P, Mohseni M, Rafiei S. | Senior Managers’ Viewpoints Toward Challenges of Implementing Clinical Governance: A National Study in Iran | 2013 | Article | International Journal of Health Policy and Management | Empirical research | Cross-sectional quantitative study |
| 75 | Schuster RJ, Cherry CO, Smith ML. | The Clinician Engagement and Education Session: Modernizing “Academic Detailing” | 2013 | Perspectives and others | American Journal of Medical Quality | Other | Commentary |
| 76 | Thomas L, Galla C. | Building a culture of safety through team training and engagement | 2013 | Article | BMJ Quality & Safety | Empirical research | Before-after study |
| 77 | Department of Health (DoH) - NHS | Effective Clinical and Financial Engagement: A best practice guide for the NHS | 2013 | Report | NHS - Department of Health | Policy document | Mixed, quali/quantitative methods |
| 78 | Stevens GW. | Engaging Employed Physicians: Reconceptualizing The Role of Collective Identification | 2014 | Book section | Annual Review of Health Care Management: Revisiting The Evolution of Health Systems Organization | Conceptual Paper | Theoretical development |
| 79 | Clark J, Nath V. | Medical engagement: A journey not an event | 2014 | Report | The King's Fund | Empirical research | Case study/ies |
| 80 | Detwiller M, Petillion W. | Change Management and Clinical Engagement: Critical Elements for a Successful Clinical Information System Implementation | 2014 | Article | CIN: Computers, Informatics, Nursing | Report | Case study/ies |
| 81 | Hartley K, Kautsch M. | Polish and UK doctors’ engagement with hospital management | 2014 | Article | International Journal of Public Sector Management | Empirical research | Qualitative study |
| 82 | Kaissi A. | Enhancing Physician Engagement: An International Perspective | 2014 | Article | International Journal of Health Services | Conceptual Paper | Review (not systematic) |
| 83 | Lee TH, Cosgrove T. | Engaging Doctors in the Health Care Revolution | 2014 | Article | Harvard Business Review | Position Paper | Commentary |
| 84 | Mache S, Vitzthum K, Klapp BF, Danzer G. | Surgeons' work engagement: Influencing factors and relations to job and life satisfaction | 2014 | Article | The Surgeon | Empirical research | Cross-sectional quantitative study |
| 85 | Milliken AD. | Physician engagement: A necessary but reciprocal process | 2014 | Perspectives and others | CMAJ: Canadian Medical Association Journal | Other | Commentary |
| 86 | Parand A, Dopson S, Renz A, Vincent C. | The role of hospital managers in quality and patient safety: a systematic review | 2014 | Article | BMJ Open | Conceptual Paper | Systematic review |
| 87 | Riches E, Robson B. | Clinical engagement: Improving healthcare together | 2014 | Article | Scottish Medical Journal | Policy document | Policy Insight |
| 88 | Saleeby E, Holschneider CH, Singhal R | Paradigm shifts: using a partecipatory leadership process to redesign health systems | 2014 | Article | Current Opinion in Obstetrics & Gynecology | Report | Case study/ies |
| 89 | Kreindler SA, Larson BK, Wu FM, Gbemudu JN, Carluzzo KL, Struthers A, Van Citters AD, Shortell SM, Nelson EC, Fisher ES. | The rules of engagement: physician engagement strategies in intergroup contexts | 2014 | Article | Journal of Health Organization and Management | Empirical research | Case study/ies |
| 90 | Spaulding A, Gamm L, Menser T. | Physician Engagement: Strategic Considerations among Leaders at a Major Health System. | 2014 | Article | Hospital Topics | Empirical research | Qualitative study |
| 91 | Studer Q, Hagins M, Jr., Cochrane BS. | The Power of Engagement: Creating the Culture That Gets Your Staff Aligned and Invested | 2014 | Article | Healthcare Management Forum | Conceptual Paper | Theoretical development |
| 92 | Vath R. | Engaging physician leaders for improved outcomes | 2014 | Perspectives and others | Health progress | Report | Case study/ies |
| 93 | Jarousse LA. | Physician engagement (getting your docs on board!) | 2014 | Article | Hospitals & Health Networks | Report | Critical debate |
| 94 | Klugman R, Gitkind MJ, Walsh KE. | The Physician Quality Officer Model: 5-Year Follow-up | 2015 | Article | American Journal of Medical Quality | Report | Case study/ies |
| 95 | Morrissey, J. | The Importance of Physician Leadership | 2015 | Perspectives and others | Trustee | Other | Critical debate |
| 96 | Beckham JD, Berry LL, Feussner JR, Trastek VF. | Strategic teamwork in health care: the essential role of physicians | 2015 | Perspectives and others | Physician Leadership Journal | Position Paper | Commentary |
| 97 | Byrnes J. | Great physician engagement is key to great quality | 2015 | Perspectives and others | Physician Leadership Journal | Position Paper | Commentary |
| 98 | Govender T. | Physician Engagement and Documentation Excellence | 2015 | Perspectives and others | Journal of Health Care Compliance | Position Paper | Commentary |
| 99 | Greysen SR, Detsky AS. | Solving the puzzle of posthospital recovery: What is the role of the individual physician? | 2015 | Article | Journal of Hospital Medicine | Position Paper | Commentary |
| 100 | Rosenstein AH. | Strategies to Enhance Physician Engagement | 2015 | Article | The Journal of Medical Practice Management | Position Paper | Commentary |
| 101 | Rosentein A. | Letter to the Editor: True physician engagement | 2015 | Perspectives and others | Hospitals & Health Networks | Letter | Letter to the editor |
| 102 | Sonnenberg M. | Chief Medical Officer: Changing Roles and Skill Sets | 2015 | Article | Physician Leadership Journal | Position Paper | Theoretical development |
| 103 | Spurgeon P, Long P, Clark J, Daly F. | Do we need medical leadership or medical engagement? | 2015 | Article | Leadership in Health Services | Conceptual Paper | Mixed, quali/quantitative methods |
| 104 | Till A, Banerjee J, McKimm J. | Supporting the engagement of doctors in training in quality improvement and patient safety | 2015 | Article | British Journal of Hospital Medicine | Conceptual Paper | Review (not systematic) |
| 105 | Zimlich R. | Physician Engagement: a priority, not an afterthought | 2015 | Article | Managed Healthcare Executive | Other | Critical debate |
| 106 | Tsai T C, Jha A K, Gawande A A, Huckman R S, Bloom N, Sadun R. | Hospital Board And Management Practices Are Strongly Related To Hospital Performance On Clinical Quality Metrics | 2015 | Article | Health Affairs | Empirical research | Cross-sectional quantitative study |
| 107 | The American Hospital Association (AHA) | Physician Engagement: A necessary ingredient for the transformation of health care | 2016 | Report | Hospitals & Health Networks (H&HN) | Other | Critical debate |
| 108 | Donatelli D. | Creating patient-centered Supply Chain a healthy prescription. | 2016 | Perspectives and others | Healthcare Purchasing News | Position Paper | Commentary |
| 109 | Dye CF. | Selecting Physician Leaders | 2016 | Perspectives and others | Healthcare Executive | Position Paper | Commentary |
| 110 | Dye CF. | Enhancing Physician Engagement | 2016 | Perspectives and others | Healthcare Executive | Position Paper | Commentary |
| 111 | Henson JW. | Reducing Physician Burnout Through Engagement | 2016 | Perspectives and others | Journal of Healthcare Management | Conceptual Paper | Commentary |
| 112 | Jeffs L, Indar A, Harvey B, McShane J, Bookey-Bassett S, Flintoft V, Suhemat A, Maione M. | Enabling Role of Manager in Engaging Clinicians and Staff in Quality Improvement: Being Present and Flexible. | 2016 | Article | Journal of Nursing Care Quality | Empirical research | Qualitative study |
| 113 | Klajner S. | Physicians’ engagement: Medical Care Groups | 2016 | Perspectives and others | Einstein | Report | Case study/ies |
| 114 | Laws K. | Making PPIs a positive force in provider operations | 2016 | Perspectives and others | Healthcare Purchasing News | Position Paper | Commentary |
| 115 | Macinati MS, Bozzi S, Rizzo MG. | Budgetary participation and performance: The mediating effects of medical managers' job engagement and self-efficacy | 2016 | Article | Health Policy | Empirical research | Mixed, quali/quantitative methods |
| 116 | McKee AP. | Physicians Lead the Way in Transforming Healthcare. | 2016 | Perspectives and others | Frontiers of health services management | Report | Commentary |
| 117 | Pannick S, Sevdalis N, Athanasiou T. | Beyond clinical engagement: a pragmatic model for quality improvement interventions, aligning clinical and managerial priorities | 2016 | Article | BMJ Quality & Safety | Conceptual Paper | Theoretical development |
| 118 | Paranjpe P. | How to use data analytics to engage physicians | 2016 | Perspectives and others | Health Management Technology | Position Paper | Commentary |
| 119 | Strömgren M, Eriksson A, Bergman D, Dellve L. | Social capital among healthcare professionals: A prospective study of its importance for job satisfaction, work engagement and engagement in clinical improvements | 2016 | Article | International Journal of Nursing Studies | Empirical research | Longitudinal analysis |
| 120 | Swensen S, Kabcenell A, Shanafelt T. | Physician-Organization Collaboration Reduces Physician Burnout and Promotes Engagement: The Mayo Clinic Experience | 2016 | Article | Journal of Healthcare Management | Empirical research | Case study/ies |
| 121 | Dickinson H, Bismark M, Phelps G, Loh E. | Future of medical engagement | 2016 | Perspectives and others | Australian Health Review | Conceptual Paper | Review (not systematic) |
| 122 | Jorm C. | Clinician engagement: Scoping paper | 2016 | Report | Department of Health and Human Services (Victoria) | Scoping Analysis | Mixed, quali/quantitative methods |
| 123 | Shanafelt TD, Noseworthy JH. | Executive Leadership and Physician Well-being: Nine Organizational Strategies to Promote Engagement and Reduce Burnout | 2017 | Article | Mayo Clinic Proceedings | Position Paper | Case study/ies |
| 124 | Harvey LFB, Smith KA, Curlin H. | Physician Engagement in Improving Operative Supply Chain Efficiency Through Review of Surgeon Preference Cards | 2017 | Article | Journal of Minimally Invasive Gynecology | Empirical research | Qualitative study |
| 125 | Keys Y, Silverman SR, Evans J. | Identification of Tools and Techniques to Enhance Interdisciplinary Collaboration During Design and Construction Projects | 2017 | Article | Health Environments Research and Design Journal | Empirical research | Qualitative study |
| 126 | Thakrar SV, Bell D. | Sustainability and transformation plans: Translating the perspectives | 2017 | Article | British Journal of Hospital Medicine | Conceptual Paper | Commentary |
| 127 | Sondheim SE, Patel DM, Chin N, Barwis K, Werner J, Barclay A, Mattie A. | Governance Practices in an Era of Healthcare Transformation: Achieving a Successful Turnaround | 2017 | Article | Journal of Healthcare Management | Empirical research | Case study/ies |
| 128 | Waring J, Crompton A. | A ‘movement for improvement’? A qualitative study of the adoption of social movement strategies in the implementation of a quality improvement campaign | 2017 | Article | Sociology of Health & Illness | Empirical research | Case study/ies |
| 129 | Dixit SK. | Integration of importance-performance analysis into the strategy of hospitals | 2017 | Article | International Journal of Healthcare Management | Conceptual Paper | Review (not systematic) |
| 130 | Combs T, Endicott M, Kravtsova M, Mogbo C, Sezginis NC. | Impact of physician engagement on clinical documentation improvement programs | 2017 | Article | Journal of the American Health Information Management Association | Practical Brief | Commentary |
| 131 | Gupta R, Moriates C. | Swimming Upstream: Creating a Culture of High-Value Care | 2017 | Perspectives and others | Academic Medicine | Position Paper | Commentary |
| 132 | Owens K, Eggers J, Keller S, McDonald A. | The imperative of culture: A quantitative analysis of the impact of culture on workforce engagement, patient experience, physician engagement, value-based purchasing, and turnover | 2017 | Article | Journal of Healthcare Leadership | Empirical research | Cross-sectional quantitative study |
| 133 | Minhas JS, Bains MS, Hughes A, Kirtley J, Carr S. | The doctors in training committee: A mechanism for change? | 2017 | Article | British Journal of Hospital Medicine | Report | Case study/ies |
| 134 | Ishii L, Demski R, Ken Lee KH, Mustafa Z, Frank S, Wolisnky JP, Cohen D, Khanna J, Ammerman J, Khanuja HS, Unger AS, Gould L, Wachter PA, Stearns L, Werthman R, Pronovost P. | Improving healthcare value through clinical community and supply chain collaboration | 2017 | Article | Healthcare | Empirical research | Case study/ies |
| 135 | Skillman M, Cross-Barnet C, Singer RF, Ruiz S, Rotondo C, Ahn R, Snyder LP, Colligan EM, Giuriceo K, Moiduddin A. | Physician Engagement Strategies in Care Coordination: Findings from the Centers for Medicare & Medicaid Services’ Health Care Innovation Awards Program | 2017 | Article | Health Services Research | Empirical research | Qualitative study |
| 136 | Allin S, Guilcher S, Riley D, Zhang YJ. | Improving Health System Efficiency: Perspectives of Decision-Makers | 2017 | Article | Healthcare Quarterly | Empirical research | Qualitative study |
| 137 | Garvin D, Worthington J, McGuire S, Burgetz S, Forster AJ, Patey A, Gerin-Lajoie C, Turnbull J, Roth V. | Physician performance feedback in a Canadian academic center | 2017 | Article | Leadership in Health Services | Empirical research | Case study/ies |
| 138 | Dickinson H, Snelling I, Ham C, Spurgeon PC. | Are we nearly there yet? A study of the English National Health Service as professional bureaucracies | 2017 | Article | Journal of Health, Organisation and Management | Empirical research | Case study/ies |
| 139 | Spurgeon P, Clark J. | Medical Leadership: The Key to Medical Engagement and Effective Organisations, 2nd Edition | 2017 | Book | CRC Press | Conceptual Paper | Theoretical development |
| 140 | Ireri S K, Walshe K, Benson L, Mwanthi M. | A comparison of experiences, competencies and development needs of doctor managers in Kenya and the United Kingdom (UK) | 2017 | Article | The International Journal of Health Planning and Management | Empirical research | Mixed, quali/quantitative methods |
| 141 | Perreira TA, Perrier L, Prokopy M. | Hospital Physician Engagement: A Scoping Review | 2018 | Article | Medical Care | Conceptual Paper | \| Scoping review \| \| --- \| |
| 142 | PM SK, Sagayam MS, Janardhanan A, Sanjeev L. | Performance and Purchasing effects of Healthcare Supply Chain | 2018 | Conference Paper | 2018 International Conference on Advances in Computing, Communications and Informatics, ICACCI 2018 | Protocol | Mixed, quali/quantitative methods |
| 143 | Diraviam SP, Sullivan PG, Sestito JA, Nepps ME, Clapp JT, Fleisher LA. | Physician Engagement in Malpractice Risk Reduction: A UPHS Case Study | 2018 | Article | Joint Commission Journal on Quality and Patient Safety | Empirical research | Case study/ies |
| 144 | Underdahl L, Jones-Meineke T, Duthely LM. | Reframing physician engagement: An analysis of physician resilience, grit, and retention | 2018 | Article | International Journal of Healthcare Management | Conceptual Paper | Review (not systematic) |
| 145 | Wilson KM, Leeman J, Saunders B, Havens DS. | Improving physician engagement in interprofessional collaborative practice in rural emergency departments | 2018 | Article | Journal of Interprofessional Education & Practice | Empirical research | Case study/ies |
| 146 | Donaghy G, McKeever K, Flanagan C, O’Kane D, McQuillan B, Cash J, Jack C, Lundy C. | Helping doctors in training to STEP-UP: A leadership and quality improvement programme in the belfast health and social care trust | 2018 | Article | Ulster Medical Journal | Report | Mixed, quali/quantitative methods |
| 147 | Dellve L, Strömgren M, Williamsson A, Holden RJ, Eriksson A. | Health care clinicians’ engagement in organizational redesign of care processes: The importance of work and organizational conditions | 2018 | Article | Applied Ergonomics | Empirical research | Longitudinal analysis |
| 148 | Rinne ST, Rinne TJ, Olsen K, Wiener RS, Balcezak TJ, Dardani W, Elwy AR. | Hospital administrators’ perspectives on physician engagement: A qualitative study | 2018 | Article | Journal of Hospital Medicine | Empirical research | Qualitative study |
| 149 | Perreira T, Perrier L, Prokopy M, Jonker A. | Physician engagement in hospitals: A scoping review protocol | 2018 | Article | BMJ Open | Protocol | Scoping review protocol |
| 150 | Engelman D, Benjamin EM. | Physician Engagement: The “Secret Sauce” to Success in Bundled Health Care | 2018 | Perspectives and others | American Journal of Medical Quality | Conceptual Paper | Commentary |
| 151 | Saxena A, Davies M, Philippon D. | Structure of health-care dyad leadership: an organization’s experience | 2018 | Article | Leadership in Health Services | Empirical research | Mixed, quali/quantitative methods |
| 152 | Rabkin SW, Dahl M, Patterson R, Mallek N, Straatman L, Pinfold A, Charles MK, van Gaal S, Wong S, Vaghadia H. | Physician engagement: the Vancouver Medical Staff Association engagement charter | 2019 | Article | Clinical medicine | Position Paper | Theoretical development |
| 153 | Kim J., Israel E., Rao S., Aaronson E., Weilburg J., Kaafarani H., Lee J. | Reduction in pediatric gastroenterology ED visits can be sustained through physician accountability and financial incentives | 2019 | Article | The American Journal of Emergency Medicine | Empirical research | Before-after study |
| 154 | van de Riet MCP, Berghout MA, Buljac-Samardžić M, van Exel J, Hilders CGJM. | What makes an ideal hospital-based medical leader? Three views of healthcare professionals and managers: A case study | 2019 | Article | PLOS ONE | Empirical research | Mixed, quali/quantitative methods |
| 155 | Cochrane BS, Ritchie D, Lockhard D, Picciano G, King JA, Nelson B. | A culture of compassion: How timeless principles of kindness and empathy become powerful tools for confronting today’s most pressing healthcare challenges | 2019 | Article | Healthcare Management Forum | Conceptual Paper | Review (not systematic) |
| 156 | Teh J, Ahmed F. | Clinician engagement is essential for the diffusion of digital innovation | 2019 | Perspectives and others | British Journal of Hospital Medicine | Other | Editorial |
| 157 | Goyal S, Law E. | An introduction to Kaizen in health care | 2019 | Article | British Journal of Hospital Medicine | Conceptual Paper | Review (not systematic) |
| 158 | Keller EJ, Giafaglione B, Chrisman HB, Collins JD, Vogelzang RL. | The growing pains of physician-administration relationships in an academic medical center and the effects on physician engagement | 2019 | Article | PLOS ONE | Empirical research | Qualitative study |
| 159 | Jorm C, Hudson R, Wallace E. | Turning attention to clinician engagement in Victoria | 2019 | Article | Australian Health Review | Report | Review (not systematic) |
| 160 | Baathe F, Rosta J, Bringedal B, Ro KI. | How do doctors experience the interactions among professional fulfilment, organisational factors and quality of patient care? A qualitative study in a Norwegian hospital | 2019 | Article | BMJ Open | Empirical research | Qualitative study |
| 161 | Scher E, Whitehouse S, Van Harn M, Bollinger J, Stevens B, Macki K, Saoud D, Baker-Genaw K. | Does physician engagement affect satisfaction of patients or resident physicians? | 2019 | Article | Journal of Healthcare Leadership | Empirical research | Longitudinal analysis |
| 162 | Turner P. | Leadership in Healthcare. Delivering Organisational Transformation and Operational Excellence | 2019 | Book | Palgrave Macmillan | Conceptual Paper | Theoretical development |
| 163 | Yanchus NJ, Carameli KA, Ramsel D, Osatuke K. | How to make a job more than just a paycheck: Understanding physician disengagement. | 2020 | Article | Health Care Management Review | Empirical research | Qualitative study |
| 164 | Gray CF, Parvataneni HK, Bozic KJ. | Value-based Healthcare: "physician Activation": Healthcare Transformation Requires Physician Engagement and Leadership | 2020 | Perspectives and others | Clinical Orthopaedics and Related Research® | Conceptual Paper | Commentary |
| 165 | Busari JO, Onitilo AA. | Fact or fable: The truth about physician engagement and burnout | 2020 | Perspectives and others | Clinical Medicine & Research | Conceptual Paper | Commentary |
| 166 | Rao SK, Ferris TG, Hidrue MK, Lehrhoff SR, Lenz S, Heffernan J, McKee KE, Del Carmen MG. | Physician burnout, engagement, and career satisfaction in a large academic medical practice | 2020 | Article | Clinical Medicine & Research | Empirical research | Longitudinal analysis |
| 167 | Fung KKW. | Physician managers in Hong Kong public hospitals | 2020 | Article | Journal of Health Organization and Management | Empirical research | Qualitative study |
| 168 | Goitein L. | Clinician-Directed Performance Improvement: Moving Beyond Externally Mandated Metrics | 2020 | Article | Health Affairs | Empirical research | Case study/ies |
| 169 | Scott IA, Kallie J, Gavrilidis A. | Achieving greater clinician engagement and impact in health care improvement: a neglected imperative | 2020 | Article | Medical Journal of Australia | Conceptual Paper | Review (not systematic) |
| 170 | Vilendrer SM, Asch SM, Anzai Y, Maggio P. | An Incentive to Innovate: Improving Health Care Value and Restoring Physician Autonomy through Physician-Directed Reinvestment | 2020 | Article | Academic Medicine | Empirical research | Case study/ies |
| 171 | Grady C, Han H, Roberts L, Van Iersel R. | Effectively engaging physicians in system change | 2020 | Article | Healthcare Management Forum | Empirical research | Longitudinal analysis |
| 172 | Savage M, Savage C, Brommels M, Mazzocato P. | Medical leadership: boon or barrier to organisational performance? A thematic synthesis of the literature | 2020 | Article | BMJ Open | Conceptual Paper | Systematic review |
| 173 | Price T, Tredinnick-Rowe J, Walshe K, Tazzyman A, Ferguson J, Boyd A, Archer J, Bryce M. | Reviving clinical governance? A qualitative study of the impact of professional regulatory reform on clinical governance in healthcare organisations in England | 2020 | Article | Health Policy | Empirical research | Qualitative study |
| 174 | Melder A, Robinson T, McLoughlin I, Iedema R, Teede H. | An overview of healthcare improvement: unpacking the complexity for clinicians and managers in a learning health system | 2020 | Article | Internal Medicine Journal | Conceptual Paper | Review (not systematic) |
