# Supplementary material for: Exploring physician engagement in health care organizations: a scoping review
Source: BMC Health Serv Res. 2023 Sep 26;23:1029. doi: 10.1186/s12913-023-09935-1 (PMC10521513; doi:10.1186/s12913-023-09935-1)
Supplement: Supplementary file 2 — Additional file 2: PRISMA-ScR checklist. [file 12913_2023_9935_MOESM2_ESM.docx]

Supplementary Material.

The implementation of the PRISMA-ScR protocol

| **SECTION** | **ITEM** | **PRISMA-ScR CHECKLIST ITEM** | **DESCRIPTION** | **REPORTED ON PAGE #** |
| --- | --- | --- | --- | --- |
| **TITLE** | | | |  |
| Title | 1 | Identify the report as a scoping review. | The title “Exploring Physician Engagement in health care organizations: a scoping review” clearly identifies the study as a “scoping review”. | 1 |
| **ABSTRACT** | | | |  |
| Structured summary | 2 | Provide a structured summary that includes (as applicable): background, objectives, eligibility criteria, sources of evidence, charting methods, results, and conclusions that relate to the review questions and objectives. | The abstract is structured and it includes detailed information on the study background, objectives, methods, results, discussion, and conclusions | 1 |
| **INTRODUCTION** | | | |  |
| Rationale | 3 | Describe the rationale for the review in the context of what is already known. Explain why the review questions/objectives lend themselves to a scoping review approach. | Sections 1 of the manuscript delivers the background against which this study was conceived, contextualizes the main study topic (clinical engagement), and spots the research focus. The combination enabled us to clearly argue the rationale of our literature review. | 3/4 |
| Objectives | 4 | Provide an explicit statement of the questions and objectives being addressed with reference to their key elements (e.g., population or participants, concepts, and context) or other relevant key elements used to conceptualize the review questions and/or objectives. | The research questions are carefully spotted in Section 2 “Study aims”. In particular, our review addressed the following questions:  a) How is the concept of physician engagement defined?  b) What do we know about the factors that can promote or impede it?  c) How does physician engagement relate to organizational performance, quality, and safety?  d) Which tools can be used to measure physician engagement? | 4/5 |
| **METHODS** | | | |  |
| Protocol and registration | 5 | Indicate whether a review protocol exists; state if and where it can be accessed (e.g., a Web address); and if available, provide registration information, including the registration number. | Our research protocol is carefully depicted in section 3. Even though we did not publish a research protocol in an earlier article, as this practice is uncommon in the domain of Social Sciences, we provided all relevant information to ensure the dependability and the replicability of our review. | 5/8 |
| Eligibility criteria | 6 | Specify characteristics of the sources of evidence used as eligibility criteria (e.g., years considered, language, and publication status), and provide a rationale. | The exclusion criteria which guided items’ analysis and selection are duly described in subsection “3.2 Step 2: Exclusion criteria”, which clearly identifies the rules agreed by the authors to screen the items which were extracted from bibliographic search. | 6/7 |
| Information sources* | 7 | Describe all information sources in the search (e.g., databases with dates of coverage and contact with authors to identify additional sources), as well as the date the most recent search was executed. | We spotted the source for bibliographic search in subsection “3.1 Step 1: literature search”. We justified our research strategy considering the aims which triggered our scoping review | 5/6 |
| Search | 8 | Present the full electronic search strategy for at least 1 database, including any limits used, such that it could be repeated. | The search string is presented in subsection “3.1 Step 1: literature search”. We supported its consistency with the study aims | 6 |
| Selection of sources of evidence† | 9 | State the process for selecting sources of evidence (i.e., screening and eligibility) included in the scoping review. | The whole process of items’ screening and analysis is presented in subsections “3.2 Step 2: Exclusion criteria” and “3.3. Step 3: Record analysis”. We also added a flowchart which graphically depicts the process of items’ analysis and selection. | 6/8 |
| Data charting process‡ | 10 | Describe the methods of charting data from the included sources of evidence (e.g., calibrated forms or forms that have been tested by the team before their use, and whether data charting was done independently or in duplicate) and any processes for obtaining and confirming data from investigators. | We arranged a homogeneous and consistent approach to examine the items which were obtained from database search. We used a tailored electronic worksheet to align the analysis accomplished by the authors. Any effort was made to achieve consistency in the individual analysis implemented by the authors. The majority rule was followed to legitimize inclusion/exclusion decisions. | 7/8 |
| Data items | 11 | List and define all variables for which data were sought and any assumptions and simplifications made. | All criteria used to investigate the items which were obtained from bibliographic search are reported in subsection “3.3. Step 3: Record analysis”. Variables have been duly justified in light of the study purposes. | 7/8 |
| Critical appraisal of individual sources of evidence§ | 12 | If done, provide a rationale for conducting a critical appraisal of included sources of evidence; describe the methods used and how this information was used in any data synthesis (if appropriate). | Although we did not implement a critical appraisal of included sources, we carefully assessed each items which was included in the analysis, sorting out records which did not effectively contribute in advancing the scientific knowledge about clinical engagement (see exclusion criteria) | 6/7 |
| Synthesis of results | 13 | Describe the methods of handling and summarizing the data that were charted. | We carefully depicted the whole process of items selection and analysis in a flowchart, which is currently included at the end of the methods section. | 8 |
| **RESULTS** | | | |  |
| Selection of sources of evidence | 14 | Give numbers of sources of evidence screened, assessed for eligibility, and included in the review, with reasons for exclusions at each stage, ideally using a flow diagram. | In order to achieve an increased clarity in the presentation of our research methodology, we included a flowchart depicting the whole process of items’ collection, analysis, and selection at the end of the methods’ section. The numbers and the reasons for exclusion at each stage are provided in the flowchart and in sub-section “3.3. Step 3: Record analysis”. | 7/8 |
| Characteristics of sources of evidence | 15 | For each source of evidence, present characteristics for which data were charted and provide the citations. | The first subsection of the study results (“4.1 Overview”) delivers an overview of the items which were included in the literature review. | 8/11 |
| Critical appraisal within sources of evidence | 16 | If done, present data on critical appraisal of included sources of evidence (see item 12). | Whilst we did not conduct a thorough appraisal of included sources, we carefully screened the attributes of the items included in our scoping review, providing some descriptive information which are currently reported in subsection “4.1 Overview”. | 8/11 |
| Results of individual sources of evidence | 17 | For each included source of evidence, present the relevant data that were charted that relate to the review questions and objectives. | Throughout the report of the study findings, we made an effort to clearly and transparently report the key evidence which were extracted from the literature. | 11/22 |
| Synthesis of results | 18 | Summarize and/or present the charting results as they relate to the review questions and objectives. | We used both a narrative approach and tables and figures to deliver a comprehensive account of the study results. Furthermore, the subsections in which the results’ section is articulated are framed consistently with the study aims. | 11/22 |
| **DISCUSSION** | | | |  |
| Summary of evidence | 19 | Summarize the main results (including an overview of concepts, themes, and types of evidence available), link to the review questions and objectives, and consider the relevance to key groups. | We summarize the main evidence collected from our literature review in the discussion section, linking them to the main questions which inspired our research and providing the readers with some intriguing insights to make sense of the state of the art in the domain of physician engagement. | 22/24 |
| Limitations | 20 | Discuss the limitations of the scoping review process. | We discussed the main limitations which affected our scoping review. This enabled us to identify the shortcomings of our protocol and to envision avenues for further development. | 24/25 |
| Conclusions | 21 | Provide a general interpretation of the results with respect to the review questions and objectives, as well as potential implications and/or next steps. | We concluded the paper with a general interpretation of the results and several avenues for next steps, coming from the analysis of the results in light of the research questions. | 25 |
| **FUNDING** | | | |  |
| Funding | 22 | Describe sources of funding for the included sources of evidence, as well as sources of funding for the scoping review. Describe the role of the funders of the scoping review. | We did not obtain any funding for conducting this research, which was self-financed by the authors. | NA |
